# Association between school bullying victimization and self-harm in a sample of Chinese children and adolescents: The mediating role of perceived social support

**DOI:** 10.3389/fpubh.2022.995546

**Published:** 2022-11-10

**Authors:** Yusan Che, Jin Lu, Die Fang, Hailiang Ran, Sifan Wang, Xuemeng Liang, Hao Sun, Junwei Peng, Lin Chen, Yuanyuan Xiao

**Affiliations:** ^1^Department of Epidemiology and Health Statistics, School of Public Health, Kunming Medical University, Kunming, China; ^2^Department of Psychiatry, The First Affiliated Hospital, Kunming Medical University, Kunming, China

**Keywords:** self-harm, bullying victimization, social support, parental support, mediation

## Abstract

**Background:**

Studies indicated that bullying victimization (BV) is an important risk factor for self-harm in children and adolescents. However, it is unclear whether perceived social support significantly mediates this association. This study aimed to examine the association between BV and self-harm, with a particular focus on the mediating role of perceived social support.

**Methods:**

A population-based cross-sectional study of 4,627 Chinese students aged from 10 to 17 years was conducted in southwestern China Yunnan province. A two-stage simple random cluster sampling method was used to choose study subjects. The adjusted associations between school BV, perceived social support, and self-harm were examined by using the multivariate logistic regression models. The mediation of perceived social support in the association between BV and self-harm was evaluated by using a path model.

**Results:**

After controlling potential covariates, BV was associated with a prevalence of increased self-harm, with an adjusted odds ratio of 1.90 (95% CI: 1.57, 2.32). Among all sources of perceived social support, only parental support played a prominent mediating role in the association between BV and self-harm, accounting for 20.73% of the explained variance. The mediation of parental support was comparable between boys and girls. As for different types of bullying victimization, path analyses indicated that only the association between physical victimization and self-harm was significantly mediated by parental support.

**Conclusion:**

Our study results highlighted the promising interventional benefit of parental support in BV-associated self-harm risk for children and adolescents. For victims of bullying, especially physical bullying, promoting parental support might be effective in reducing self-harm risk. Longitudinal studies are warranted to further corroborate these findings.

## Introduction

Self-harm is a non-fatal behavior that is intentionally initiated by a person, such as hurting the body or ingesting a toxic substance or object, to cause harm to oneself (1). Compared with other age groups, children and adolescents are more vulnerable to self-harm (2). Globally, the prevalence of self-harm among 12 to 21 years old ranges from 3.1 to 15.5% (3). In China, the reported prevalence of self-harm can be as high as 27.6% (4). Self-harming behavior among adolescents is significantly associated with serious negative consequences including increased risk of behavioral problems, poor academic performance, social isolation, and several psychiatric disorders such as depression (5, 6). Various sociodemographic, psychological, and psychosocial factors were identified to be associated with increased self-harm risk for children and adolescents (7–9).

A previously published study revealed that school bullying victimization (BV) was related to 2.2 folds of the prevalence of self-harm in a large sample of 3,146 Chinese children and adolescents (10). Another cohort study showed that BV was associated with an increased risk of future self-harm, with a risk ratio of 3.53 (11). In addition, the combined results of a meta-analysis also suggested that BV was a prominent risk factor for self-harm (12). Bullying is usually defined as deliberate, repetitive, and harmful aggressive behavior that results from an imbalance of power between the abuser and the victim (13). Traditional bullying can be categorized into three common subtypes: physical, verbal, and relational (14). BV is common among adolescents, with a reported prevalence of 18.3% (15). It is also a serious problem in China, where about half of the students reported being bullied throughout their lives (16, 17). BV is significantly associated with mental health problems, such as depression and anxiety, all are identified risk factors of self-harm (18, 19). For school children, direct intervention in BV is impractical, either because of its hidden nature, as one-fourth of victims chose not to tell anyone (20), or the limited effect of resource-intensive whole-school anti-bullying programs (21). Therefore, finding and intervening on variables that mediate the association between BV and self-harm could be a beneficial strategy in reducing BV-related self-harm.

Social support indicates the perceived and actual help one can obtain from family, friends, and significant others (22). Researchers have found that higher perceived social support (23, 24), especially parental support (25), was related to lower self-harm prevalence among adolescents. Moreover, a study of 5,012 Chinese university students reported that BV was associated with a lower level of perceived social support (26). Some researchers further explored the relationship between BV and different types of perceived social support and found that BV was inversely associated with parental support (27) and teenagers who were bullied received less support from parents and peers (28). The above evidence suggests that perceived social support may play a mediating role in the association between BV and self-harm. However, this hypothesis needs to be thoroughly discussed, especially among children and adolescents from Eastern countries. Previous studies suggested that Eastern and Western cultures may impose different influences on the self-harm behaviors of the youths, as Eastern parents have more influence on adolescents' behaviors, and in collectivistic Eastern cultures, self-harm could be perceived as more negative, and adolescents may worry more about others' reaction to their self-harming behavior (29–31).

The main purpose of this study was to evaluate this suspected mediation by perceived social support in the association between BV and self-harm in a large representative sample of Chinese children and adolescents. The following two hypotheses were tested:

*Hypothesis 1:* Perceived social support in general significantly mediates the association between BV and self-harm.

*Hypothesis 2:* Different sources of perceived social support present discordant mediation in this association.

## Materials and methods

### Participants and procedure

This cross-sectional survey was conducted from 19 October to 3 November 2020. The participants were determined by using a two-stage simple random clustering sampling method in Kaiyuan city, Honghe Hani and Yi autonomous prefecture, Yunnan province, China. The sample size was preliminarily calculated by using the formula for the simple random sampling method. We set a conservative self-harm prevalence of 10%, with an acceptable error of 1.3% and an estimated effective response rate of 90%, which yielded a preliminarily calculated sample size of 2,274. Since the sampling error of multi-stage cluster sampling is greater than that of simple random sampling, we used a conservative design-effect (Deff) value of 2 for further adjustment, so the final calculated sample size was 4,548. All eligible participants were asked to complete self-administered paper questionnaires independently, taking about 45 min to finish. Respondents were required to sit apart when filling in the questionnaire, any communication between the respondents will be immediately stopped by quality control personnel deployed at the site, who will also check the completeness and logic of the filled questionnaires. Because participants were all under 18 years old, parents or legal guardians provided signed informed consent prior to the survey. Other details of survey implementation can be referred to in our previous publication (32).

Eligible participants were those aged between 10 and 18. We set a lower age limit of 10 because we simultaneously measured suicidal ideation and behaviors in this survey, only children aged 10 and above can fully understand the concept and consequences of suicide (33). The following exclusion criteria were further used: (1) illiteracy; (2) cognitive disorders; (3) serious physical illnesses. The final sample size for data analyses in this study was 4,627, aged between 10 and 17 years; of which, 2,283 were boys (49.34%). The study protocol was reviewed and approved by the Ethics Review Committee of Kunming Medical University (No. KMMU2020MEC047).

### Measures

A comprehensive questionnaire that contains multiple modules was used to collect information from the participants. The current study used the following parts: basic information (such as demographics, family and socioeconomic status, etc.), perceived social support, BV, self-harm, depression, and anxiety.

#### Bullying victimization

The Chinese version of the Olweus Bully/Victim Questionnaire (OBVQ) was used to measure the traditional bullying (physical, verbal, and relation) involvement of the respondents (34). Each type of BV is measured by two separate questions, and all the questions have identical five responses indicating the frequency (never happened, once or twice, two or three times a month, once a week, and several times a week) of a particular BV behavior. In this study, we used widely accepted criteria to define BV: if a respondent reported “two or three times a month” or was more frequently bullied, this person will be labeled as a victim (35). During data analysis, school BV had been included as a binary variable.

#### Perceived social support

Perceived social support was estimated by using the Chinese version of the Child and Adolescent Perceived Social Support Scale (CASSS) (36). CASSS can be divided into four parts; each part measures perceived social support from a certain source (parents, teachers, classmates, and friends). One module contains 10 questions, with five-point Likert-style responses (scores ranging from 1 to 5). The lowest and the highest combined scores for each module are 10 and 50, respectively, with a higher score indicating better perceived social support of that source. Considering there are no recommended cut-offs for the four parts of CASSS, we used the medians of the combined scores to dichotomize study subjects. The Cronbach's α of CASSS for the current analytical sample is 0.91 (Bootstrap 95% CI: 0.91, 0.92).

#### Self-harm

The Modified Adolescent Self-harm Scale (MASELF-HARMS) developed by Feng was used to measure the lifetime frequency and severity of the 18 most common self-harm behaviors among Chinese adolescents (37). Self-harm frequency is measured as follows: never, once, twice to four times, five times, and more. In the current study, respondents who reported any self-harm behaviors were classified as self-harmers.

#### Depression and anxiety

The depression scale PHQ-9 contains nine questions, each with four options, with a maximum score of 27. We used a cut-off of 4 for PHQ-9 to define depressive symptoms (PHQ-9>4) (38). The Cronbach's α of PHQ-9 for the current analytical sample is 0.88 (Bootstrap 95% CI: 0.88, 0.89). The Anxiety Scale (GAD-7) has 7 items with a maximum total score of 21. A cut-off of 4 was applied to define anxiety symptoms (GAD-7>4) (39). The Cronbach's α of GAD-7 for the current analytical sample is 0.91 (Bootstrap 95% CI: 0.90, 0.91).

### Statistical analyses

The characteristics of the respondents were described by using descriptive statistics. Univariate and multivariate logistic regression models were applied to estimate the crude and adjusted associations between school BV, perceived social support, and self-harm. A path model was adopted to verify the suspected mediation of perceived social support in the association between school BV and self-harm. All statistical analyses were performed by using the R software (Version 4.0.2), and the sampling design was adjusted for throughout by using survey data-related packages. The path analysis was performed by using the “Lavaan” package; the comparative fit index (CFI), root mean square error of approximation (RMSEA), and standardized root mean square residual (SRMR) were used to measure model fit, with optimal cut-off values set as > 0.90, < 0.05, and < 0.08, respectively (40).

## Results

### Demographic information

A total of 4,858 students participated in the survey; of which, 126 were subsequently excluded as they did not meet the age criteria between 10 and 18 years, 11 respondents were removed because of missing data, and 94 respondents were further removed because of being involved in school bullying as bullies or bully-victims, not pure victims. Therefore 4,627 students were included in the final analysis, with an effective response rate of 95.24%. Among them, 2,283 were boys (49.34%), and ethnical minorities accounted for 72.44%. The mean age was 12.98 years with a standard error of 0.41. A total of 594 students reported being bullied, with a BV prevalence of 12.84%, and 1,787 reported self-harm behavior, accounting for 38.62%. Other characteristics of the respondents are summarized in Table 1.

**Table 1:** Characteristics of the participants (*N* = 4,627).

**Characteristics**	***N* (%)**	**Mean (SE)/median (IQR)**
Sex		
Variables	Univariate model	Multivariate model
Boys	2,283 (49.34)	
Girls	2,344 (50.66)	
Ethnicity		
Han majority	1,275 (27.56)	
Minorities	3,352 (72.44)	
Age		12.98 (0.41)
Grade		
Primary school	1,570 (33.93)	
Middle school	2,490 (53.81)	
High school	567 (12.25)	
Father's age		41.57 (0.46)
Mother's age		39.01 (0.48)
Left-behind children	
Yes	867 (18.74)	
No	3,760 (81.26)	
Perceived social support source	
Parents		37 (9.0)
Teacher		42 (8.0)
Classmates		37 (9.0)
Friends		39 (9.0)
Bullying victimization	
Yes	594 (12.84)	
No	4,033 (87.16)	
Type of bullying victimization	
Physical	149 (3.22)	
Verbal	471 (10.18)	
Relational	241 (5.21)	
Self-harm behaviors	
Any	1,787 (38.62)	
No	2,840 (61.38)	

### Associations between BV, perceived social support, and self-harm

**Figure 1:**
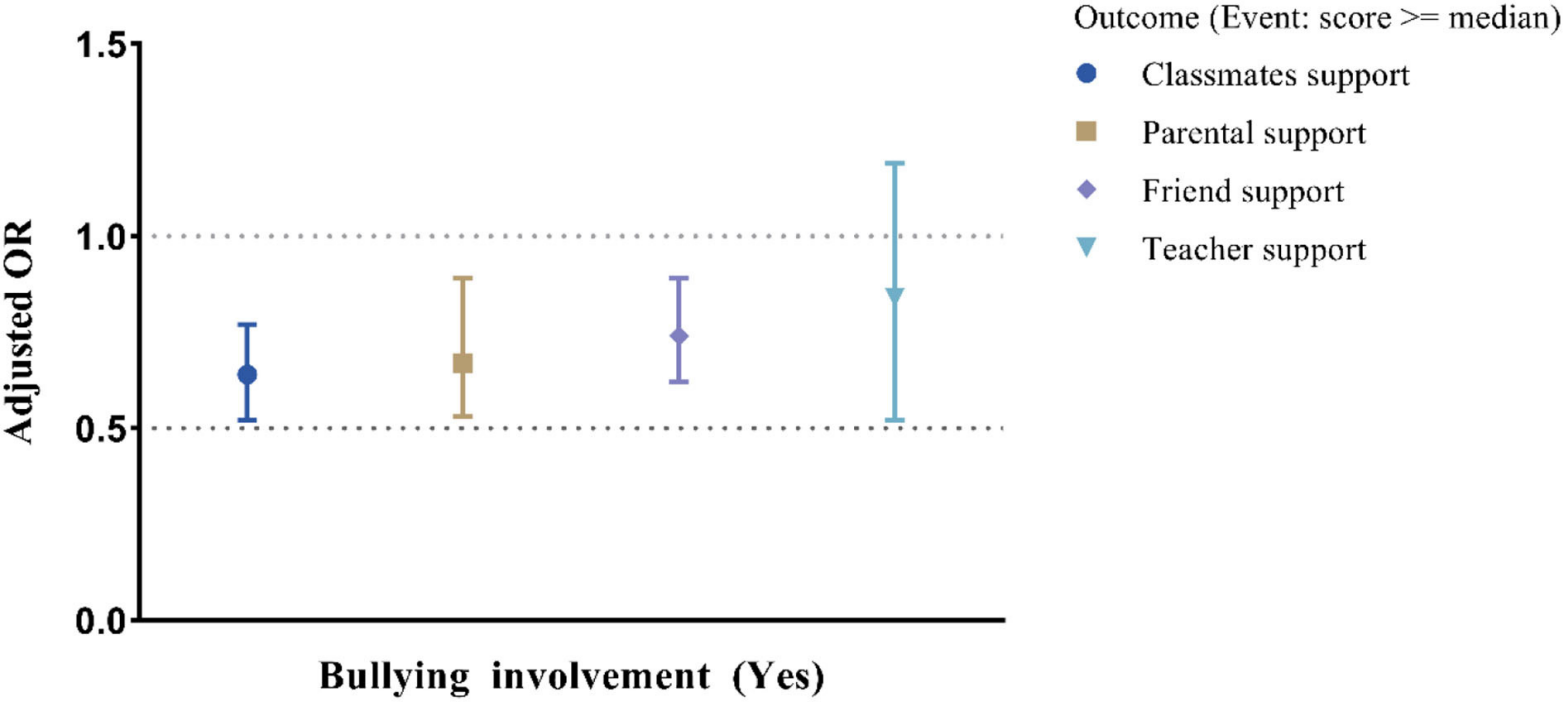
Adjusted ORs with 95% CIs for the associations between school bullying victimization (BV) and different sources of perceived social support.

The crude and adjusted associations between the three key variables are displayed in Table 2. In the multivariate model, after controlling for potential covariates, among the four sources of perceived social support, only parental support was significantly associated with self-harm. A higher level of parental support was related to an OR of 0.68 (95% CI: 0.56, 0.83), and BV was associated with an increased self-harm risk, with an OR of 1.90 (95% CI: 1.57, 2.32). The multivariate logistic regression model also revealed statistically significant associations between school BV and perceived social support from parents, classmates, and friends (Figure 1).

**Table 2:** Univariate and multivariate logistic regression models fitting results for associated factors of self-harm.

**Variables**	**Univariate model**	**Multivariate model**
	**Crude OR (90% CI)**	**Adjusted OR (95% CI)**
Sex (Ref: Boy): Girls	1.29 (1.08, 1.53)	1.08 (0.88, 1.33)
Age: +1 year	1.22 (1.15, 1.29)	1.00 (0.92, 1.08)
Ethnicity (Ref: Han majority): Minorities	0.98 (0.79, 1.21)	
Grade (Ref: Primary school)		
Middle school	2.75 (2.22, 3.39)	2.23 (1.55, 3.21)
High school	2.84 (2.09, 3.70)	1.73 (1.01, 2.94)
Father's age: +1 year	1.00 (0.99, 1.02)	
Mother's age: +1 year	1.01 (1.00, 1.02)	
Left behind children (Ref: No): Yes	1.37 (1.14, 1.65)	1.26 (1.05, 1.52)
Perceived social support source		
Parents (Ref: < 37): ≥37	0.49 (0.42, 0.57)	0.68 (0.56, 0.83)
Teachers (Ref: < 42): ≥42	0.58 (0.51, 0.67)	0.92 (0.78, 1.09)
Classmates (Ref: < 37): ≥37	0.66 (0.58, 0.74)	0.93 (0.81, 1.08)
Friends (Ref: < 39): ≥39	0.66 (0.58, 0.76)	0.89 (0.75, 1.05)
Bullying victimization (Ref: No): Yes	2.09 (1.85, 2.37)	1.90 (1.57, 2.32)
Anxiety (Ref: GAD-7 < 5): GAD-7 ≥ 5	4.89 (4.11, 5.83)	2.16 (1.78, 2.59)
Depression (Ref: PHQ-9 < 5): PHQ-9 ≥ 5	4.95 (4.08, 6.01)	2.49 (1.95, 3.16)

### The mediating role of parental support on the association between BV and self-harm

**Figure 2:**
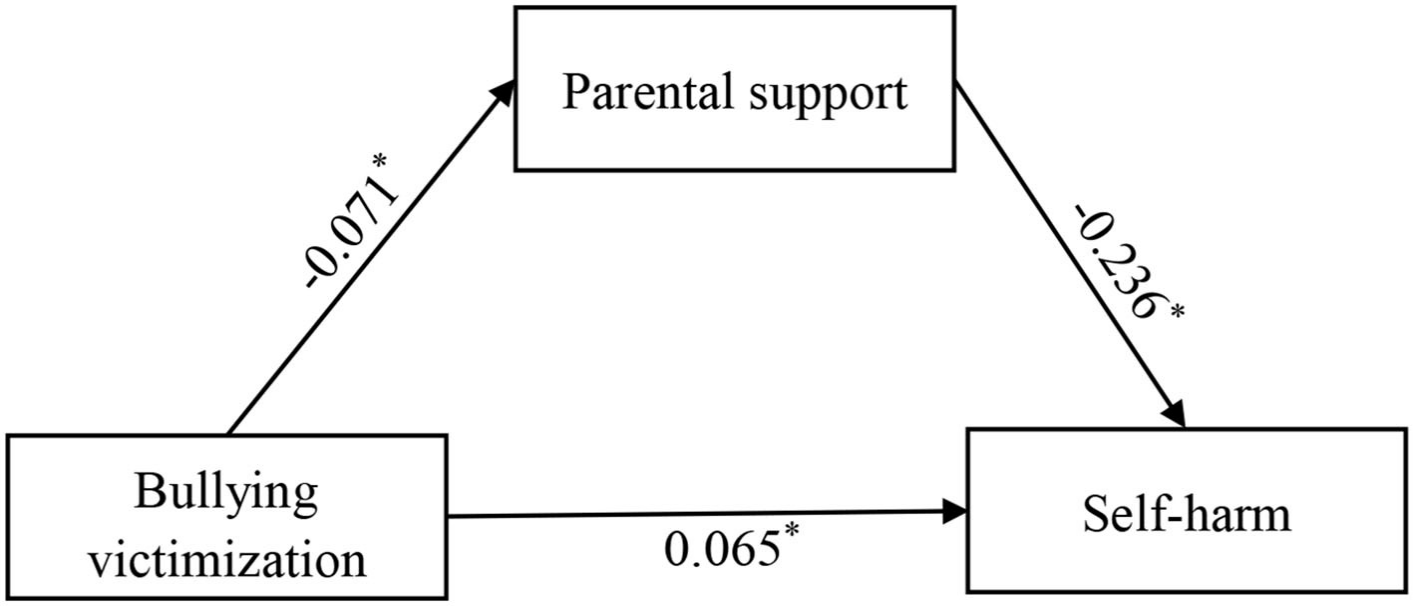
Path analysis for mediation of parental support in the association between school BV and self-harm. *p < 0.05.

The above analytical results suggest that, among the four sources of perceived social support, only parental support could be a possible mediator in the association between BV and self-harm; therefore, we further examined this hypothesis by using path analysis. The path model achieved excellent fit (CFI=1.00, RMSEA=0.00, and SRMR=0.00). The results indicated that for BV and self-harm, both their direct and indirect associations *via* parental support were statistically significant: the standardized coefficients for direct and indirect associations were (−0.071) × (−0.236)=0.017 and 0.065. The mediation by parental support accounted for [0.017/(0.017+0.065)] = 20.73% of the explained variance (Figure 2).

### The mediating role of parental support on the associations between different types of BV and self-harm

**Figure 3:**
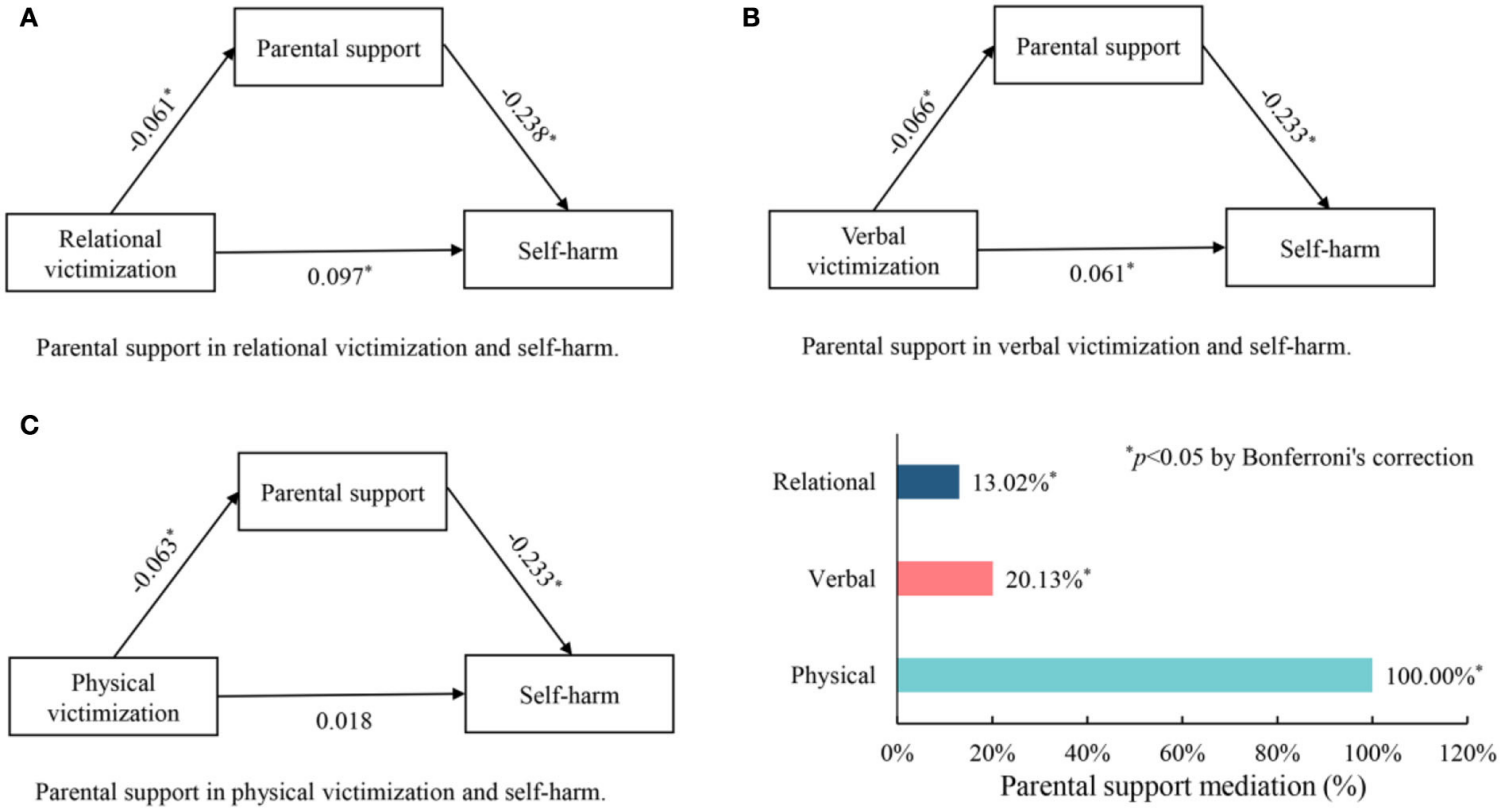
**(A–C)** Proportion of mediation by parental support for different types of BV and self-harm. *p < 0.05.

We further analyzed the mediation *via* parental support for different types of BV and self-harm, and the results are collectively shown in Figure 3. The mediation of parental support was statistically significant for all three types of BV: for relational and verbal BV, parental support mediated 13.02 and 20.13% of explained variance, respectively, whereas, for physical BV, parental support mediated 100% of explained variance, as only the indirect path *via* parental support was statistically significant. The path models achieved excellent fit with CFI = 1.00, RMSEA = 0.00, and SRMR = 0.00.

### Stratified analysis by sex

**Figure 4:**
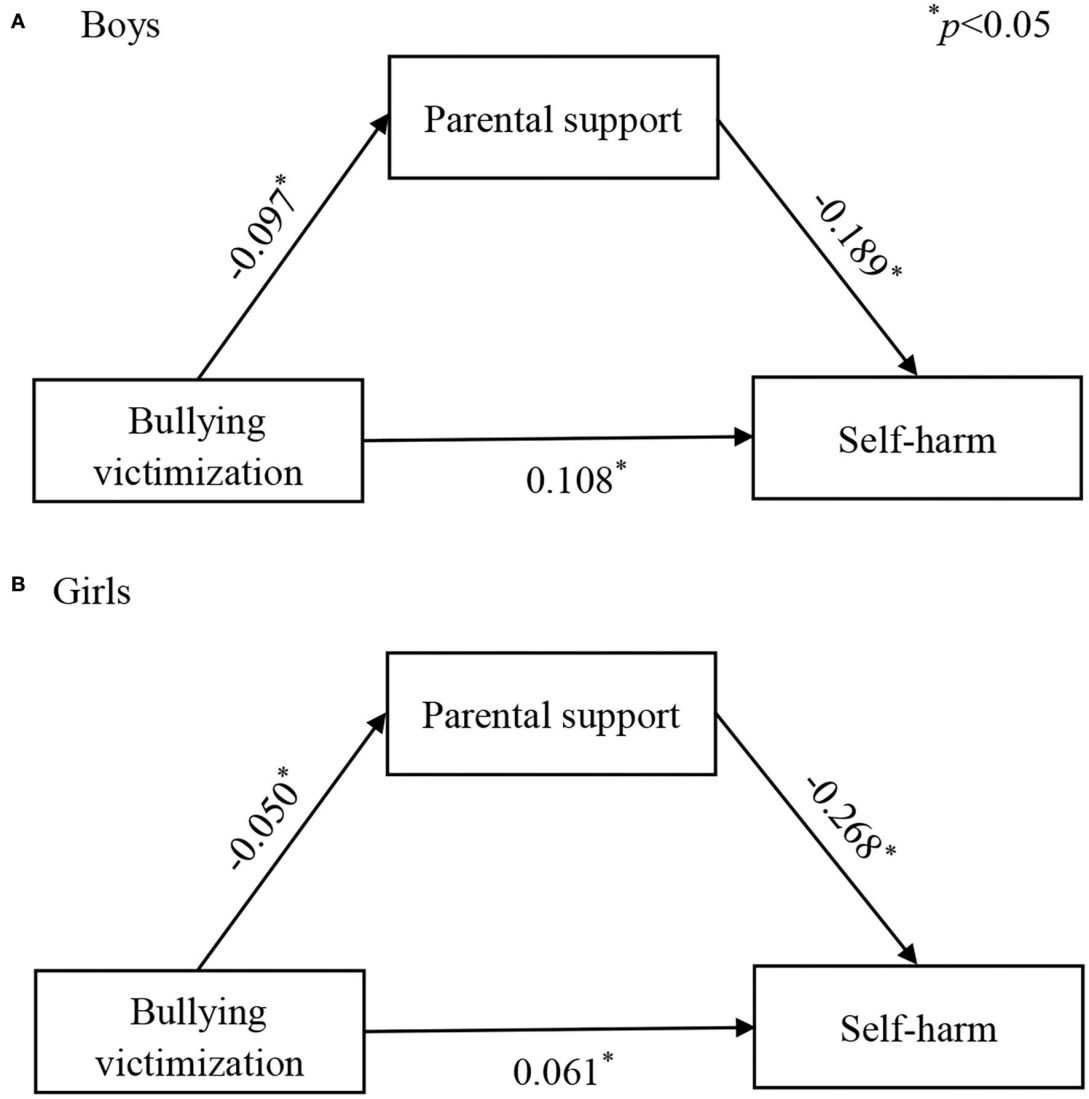
**(A,B)** Path analysis for the mediation of parental support, stratified by sex. *p < 0.05.

We examined the mediating role of parental support in the association between BV and self-harm separately across the gender groups to examine if the model performs differently across boys and girls. The results showed that the model reached excellent fit in boys (CFI = 1.00, RMSEA = 0.00, and SRMR = 0.00) and girls (CFI = 1.00, RMSEA = 0.00, and SRMR = 0.00), accounting for 14.29 and 17.57% of the explained variance, respectively (Figure 4).

## Discussion

The main purpose of this study was to investigate the mediation of perceived social support in the association between BV and self-harm in Chinese children and adolescents. We found that BV was associated with an increased risk of self-harm, which is consistent with previous studies (11, 41). Partly as anticipated, among all sources of perceived social support, only parental support presented significant mediation, mediated over one-fifth of the total association between BV and self-harm. Moreover, the mediation of parental support was comparable between boys and girls but disparate for different types of BV. The mediation of these findings not only provided new evidence on the link between BV and self-harm in children and adolescents but also shed light on the underlying mechanism of this association, especially the intervention significance of parental support in the prevention of BV-associated self-harm risk.

A previously published study revealed that parental support mediates the relationship between cyberbullying victimization and internalized symptoms (depressive symptoms and anxiety) (42). The study by DeSmet *et al*. showed that parental support mediated traditional BV and depression, anxiety, and suicidal plan (43). Besides, high family support, parental contact, and parental supervision were found protective against the suicide of bullied adolescents (44–46). Considering the close association between depression, anxiety, suicide, and self-harm, the mediation of parental support in BV and self-harm in the current study is justifiable.

Parental support directly reflects the parent-child relationship, which plays an important role in the self-harm behaviors of children and adolescents. Recently, Tao *et al*. found that a higher level of parent-child attachment was significantly associated with decreased risk of self-harm among 662 junior high school students who were also chosen from Yunnan province (47). The proposed “interpersonal theory” may well explain this positive connection between the parent-child relationship and self-harm: dysfunctional parent-child relationship predicts the development of adolescent depression (48), one of the most prominent risk factors of self-harm. Moreover, self-harm acts may also be the consequence of maladaptation, as the quality of the parent-child relationship was inversely associated with perceived stress in teenagers (49).

Although parental support was a significant mediator in the associations between all three types of BV (verbal, relational, and physical) and self-harm, it presented the strongest mediation for physical BV, mediating its entire association with self-harm. All forms of BV are associated with poorer mental health and lower levels of emotional wellbeing in adolescents (50), and among them, physical bullying was considered the most harmful (51). Compared with verbal and relational bullying, physical bullying is more likely to lead to severe suicidal ideation and suicidal attempts (52). Moreover, Peng *et al*. also found that physical BV might be a stronger risk factor for self-harm and suicide than verbal or cyber BV (53). Probably because physical BV presented a stronger association with depression than other types of BV (54), and depression is one of the most important risk factors for either self-harm or suicide (55, 56). Our findings suggest that in order to effectively prevent BV-associated self-harm risk, particularly physical BV-associated self-harm risk, strengthening parental support for bullying victims could be a practical option.

As a vital source of perceived social support for children and adolescents, it has been proven that parental support can be effectively boosted. Intervention programs, such as Resilience Triple P, Friendly Schools Friendly Families (FSFF), and Family Cognitive Behavioral Therapy (F-CBT), were found effective in enhancing parental support among children and adolescents (57–59). F-CBT was observed greater intervention effect on anxiety in children and adolescents compared to child-centered CBT (60). Moreover, it was also associated with a reduction in suicidal behavior, depression, and self-harm (61). In Resilience Triple P, a higher level of facilitative parenting was predictive to lower levels of depression and victimization of the children (62). However, these intervention programs were mostly implemented in clinical children and adolescent populations with small sample sizes; therefore, well-designed, large sample, randomized controlled studies based on the general children and adolescent population are warranted to further verify their effect.

## Limitations

Some limitations of the current study should be pointed out. The first limitation is that the cross-sectional design prevents causal inference. The second is the information bias and the problem of shared-method variance may exist as data were collected by self-reporting. However, the risk of response bias should be low, as the effective response rate exceeded 95%. The third limitation is that we looked at each type of BV separately, it is also of study interest to investigate the coexistence of multiple types of BV and the associated self-harm risk in future studies. The fourth limitation is because the OBVQ only measures traditional bullying, we did not collect information on cyberbullying, whether perceived social support also presents as a significant mediator in the association between cyberbullying and self-harm should be further investigated. Moreover, in the process of analysis, the main research variables including BV, perceived social support, and self-harm were analyzed after dichotomization, which may influence the results. Future studies with these variables analyzed in the ordinal or quantitative form need to be considered. Finally, although the sample size of this study was large, the study subjects were chosen from a certain province in China; therefore, the study results could not be generalized to other youth samples.

## Conclusion

This cross-sectional study involving 4,627 Chinese children and adolescents found that BV was significantly associated with self-harm, besides, parental support presented as a significant mediator in this association. Parental support mediated their associations with self-harm for all types of BV, especially for physical BV. Our major findings suggested that, for bullying victims, especially physical bullying victims, strengthening parental support might be effective in reducing BV-associated self-harm risk. Future studies of longitudinal design are needed to further corroborate and expand our major findings.

## Data availability statement

The raw data supporting the conclusions of this article will be made available by the authors, without undue reservation.

## Ethics statement

The studies involving human participants were reviewed and approved by the Ethics Committee of Kunming Medical University. Written informed consent to participate in this study was provided by the participants' legal guardian/next of kin.

## Author contributions

YX designed the study and critically revised the manuscript. YC, JL, DF, HR, SW, XL, HS, JP, and LC carried out the data collection. YC and YX performed data analysis. YC and JL prepared the draft manuscript. All authors critically revised the manuscript for important intellectual content.

## Funding

The study was supported by the National Natural Science Foundation of China (No. 82060601), Top Young Talents of Yunnan Ten Thousand Talents Plan (No. YNWR-QNBJ-2018-286), the Innovative Research Team of Yunnan Province (No. 202005AE160002), and Graduate Innovation Fund Project (No. 2022S007).

## Conflict of interest

The authors declare that the research was conducted in the absence of any commercial or financial relationships that could be construed as a potential conflict of interest.

## Publisher's note

All claims expressed in this article are solely those of the authors and do not necessarily represent those of their affiliated organizations, or those of the publisher, the editors and the reviewers. Any product that may be evaluated in this article, or claim that may be made by its manufacturer, is not guaranteed or endorsed by the publisher.

## Abbreviations

MASHS: The Modified version of Adolescents Self-harm Scale
OBVQ: Olweus Bully/Victim Questionnaire
CASSS: Child and Adolescent Perceived social support Scale
PHQ-9: The Patient Health Questionnaire-9
GAD-7: The Generalized Anxiety
BV: Bullying victimization.
